# Supplementary material for: Reconciling Mining with the Conservation of Cave Biodiversity: A Quantitative Baseline to Help Establish Conservation Priorities
Source: PLoS One. 2016 Dec 20;11(12):e0168348. doi: 10.1371/journal.pone.0168348 (PMC5173368; doi:10.1371/journal.pone.0168348)
Supplement: S1 Dataset — (ZIP) [file pone.0168348.s002.zip › Taxa/Serra Norte/SN_2013/N4WS/Lista N4WS-43.pdf]

CAVIDADE N4WS-0043

Classe	Ordem	Fam/Outros	Gên/Outros	Espécie	S-U	U-S	Zon.
Arachnida	Araneae	Ochyroceratidae		jovem		X	E
Arachnida	Araneae	Theridiosomatidae	*Plato*	sp.1	X		E
Arachnida	Opiliones	Sclerosomatidae	*Prionostema*	sp.1		X	E
Arachnida	Pseudoscorpiones	Chernetidae	*Spelaeochernes*	sp.1	X	X	E
Diplopoda	Polydesmida	Cyrtodesmidae		sp.1		X	E
Diplopoda	Polydesmida	Pyrgodesmidae		sp.1	X		E
Entognatha	Collembola	Paronellidae		sp.1	X		E
Insecta	Coleoptera	Carabidae		jovem	X		E
Insecta	Diptera	Ceratopogonidae		sp.	X		E
Insecta	Hymenoptera	Formicidae	*Apterostigma*	sp.1	X		E
Insecta	Hymenoptera	Formicidae	*Camponotus*	*atriceps*		1	E
Insecta	Hymenoptera	Formicidae	*Gnamptogenys*	*striatula*		X	E
Insecta	Orthoptera	Phalangopsidae	*Paraclodes*	sp.	3	1	E
Insecta	Orthoptera	Phalangopsidae		sp.1		1	E
Insecta	Psocoptera	Psocomorpha		jovem		X	E
Insecta	Psocoptera	Psyllipsocidae	*Psyllipsocus*	sp.3		X	E
